# Bacterial communities in the gut of wild and mass-reared *Zeugodacus cucurbitae* and *Bactrocera dorsalis* revealed by metagenomic sequencing

**DOI:** 10.1186/s12866-019-1647-8

**Published:** 2019-12-24

**Authors:** Ashok B. Hadapad, Suresh K. G. Shettigar, Ramesh S. Hire

**Affiliations:** 1 Nuclear Agriculture & Biotechnology Division, Bhabha Atomic Research Centre, Trombay, Mumbai, 400 085 India; 2 Cytogenetics and Molecular Genetics Section, Pathology Unit, Medical Division, Bhabha Atomic Research Centre, Trombay, Mumbai, 400 085 India; 3 Homi Bhabha National Institute (HBNI), Training School Complex, Anushaktinagar, Mumbai, 400 094 India

**Keywords:** Gut bacteria, Metagenomic sequencing, *Bactrocera* sp., Mass rearing, Bacterial endosymbionts, SIT

## Abstract

**Background:**

Insect pests belonging to genus Bactrocera sp. (Diptera: Tephritidae) pose major biotic stress on various fruits and vegetable crops around the world. Zeugodacus and Bactrocera sp. are associated with diverse bacterial communities which play an important role in the fitness of sterile insects. The wild populations of melon fly, Zeugodacus cucurbitae (Coquillett) and Oriental fruit fly, *Bactrocera dorsalis* (Hendel) were collected from pumpkin and mango fields, respectively. The laboratory populations of Z. cucurbitae and *B. dorsalis* were mass-reared on bottle gourd and sweet banana, respectively. Bacterial communities present in the gut of wild and mass-reared mature (~ 12 days old) and newly emerged (< 1 h after emergence) male and female adults of *Z. cucurbitae* and *B. dorsalis* were assessed. We used Illumina HiSeq next-generation sequencing of *16S rRNA* gene to profile the gut bacterial communities of wild and mass-reared mature and newly emerged *Z. cucurbitae* and *B. dorsalis* adults.

**Results:**

We found diverse bacterial composition in the gut of wild and mass-reared *Z. cucurbitae* (ZC) and *B. dorsalis* (BD) with varied relative abundance. Few taxonomic groups were common to both the species. The most dominant phyla in all samples of *Z. cucurbitae* and *B. dorsalis* adults were Actinobacteria, Bacteroidetes, Firmicutes and Proteobacteria. The phylum Proteobacteria occurred more in wild *Z. cucurbitae* (~ 87.72%) and *B. dorsalis* (~ 83.87%) as compared to mass-reared *Z. cucurbitae* (64.15%) and *B. dorsalis* (~ 80.96%). Higher relative abundance of Phylum Firmicutes was observed in mass-reared fruit fly than wild adults. Cyanobacteria/Chloroplast and Actinobacteria were also present with very low relative abundance in both wild as well as mass-reared melon fly and Oriental fruit fly. Enterobacteriaceae (61.21%) was dominant family in the gut of both wild and mass-reared adults. *Providencia* and *Lactococcus* were dominant genera with varied relative abundance in wild as well as in mass-reared mature and newly emerged fruit fly adults of both species. Some of the genera like *Morganella* and *Serratia* were only detected in mass-reared mature and newly emerged *Z. cucurbitae* and *B. dorsalis* adults. Principal Coordinate Analysis (PCoA) showed that fruit fly adult samples were grouped based on species and age of the adults while no grouping was observed on the basis of sex of the adult fruit fly.

**Conclusions:**

The gut bacterial communities associated with wild and mass-reared mature and newly emerged adults of *Z. cucurbitae* and *B. dorsalis* showed variation that depends on species and age of the insects. Understanding the gut microbiota of wild and mass-reared *Z. cucurbitae* and *B. dorsalis* using high throughput technology will help to illustrate microbial diversity and this information could be used to develop efficient mass-rearing protocols for successful implementation of sterile insect technique (SIT).

## Background

Fruit flies (Diptera: Tephritidae) are the economically important insect pest species and are responsible for damaging agricultural and horticultural crops. Tephritids are distributed in temperate, tropical and subtropical regions of the world [1]. The melon fly, *Zeugodacus cucurbitae* (Coquillett) and the Oriental fruit fly, *Bactrocera dorsalis* (Hendel) are the major insect pests of fruits and vegetables across Asia, Africa, Australia, and the South Pacific [1–3]. These fruit flies can cause huge economic losses in India to fruits and vegetables which vary from 30 to 100% depending upon the crop and season [2, 4]. Due to their vast adaptability, high reproduction potential and invasion capacity, tephritids have been subject of worldwide pest management programmes. The sterile insect technique (SIT) is an environment friendly, species-specific method of pest control and has been successfully implemented against various insect pests control including fruit flies [5, 6]. It has been observed that fitness of sterile males is linked with their gut microbiota [7–11]. Irradiation affects the gut microbiota and various strategies have been implemented to augment the gut microbiota using probiotics to regain the fitness of sterile insects [7–11].

Microorganisms inhabiting the intestinal tract of insects play an important role in nutrition, development, survival, resistance to pathogens, and reproduction of the host [8, 12–14]. The gut microbial composition may vary from insect to insect due to their different feeding habits [12, 13]. Different species of bacteria have been isolated and identified from the gut of various fruit fly species including *Bactrocera* species [15–20] and mainly belong to families Enterobacteriaceae, Bacillaceae, Pseudomonadaceae, Streptococcaceae, Micrococcaceae. Recently, it has been documented in a related species, *Ceratitis capitata* Wied., that supplementation of adult diet with certain gut bacteria enhances mating competitiveness, longevity, flight ability, improved pupal and adult productivity [8–11]. This will certainly help in successful implementation of SIT programme. Moreover, some gut bacteria and their supernatants have also been found to be promising in attracting fruit fly adults [18, 19, 21, 22]. Further, understanding gut bacterial diversity will facilitate in identification of important bacteria and will provide context to the differences in gut microbial communities between populations [20].

Culture dependent approaches have been employed for isolation and characterization of gut microbes in different Tephritidae species and these studies have revealed significant microbial diversity [8, 16, 18, 19, 23–25]. Molecular approaches such as *16S rRNA* gene analysis, DNA fingerprinting, Denaturing gradient gel electrophoresis (DGGE) and oligonucleotide probe-based hybridization techniques are enabling accurate identification of microbial communities of insects [8, 12, 13, 15, 17, 19, 23, 26]. However, insect gut harbor vast number of unculturable bacteria [27] which play an important role in biology of insects [12]. High throughput technologies with bacterial *16S rRNA* gene have been applied to analyze various insect’s gut microbiomes [20, 28–34].

The composition and diversity of the bacterial communities present in the gut of certain tephritids are not clearly illustrated. It has been observed that domestication, mass-rearing, diet, colony management and irradiation affect the insect gut microbiota [8, 20] which in turn impact the quality of mass-reared fruit flies used in SIT programs [8, 9, 11, 33], in addition, mass-reared fruit flies under laboratory conditions are not exposed to the natural microorganisms present in the environment. Hence, we studied the bacterial diversity and composition in midgut of wild as well as mass-reared *Z. cucurbitae* and *B. dorsalis* populations. We used Illumina HiSeq next-generation sequencing of *16S rRNA* gene to describe the bacterial communities in wild (male and female) and mass-reared mature (~ 12 days old) and newly emerged (< 1 h after emergence) *Z. cucurbitae* and *B. dorsalis* male and female adults. We found that the gut bacterial communities associated with wild and mass-reared mature and newly emerged adults of *Z. cucurbitae* and *B. dorsalis* showed variation and depends on species and age of the insects.

## Results

### 16S rRNA gene sequence reads

**Fig. 1:**
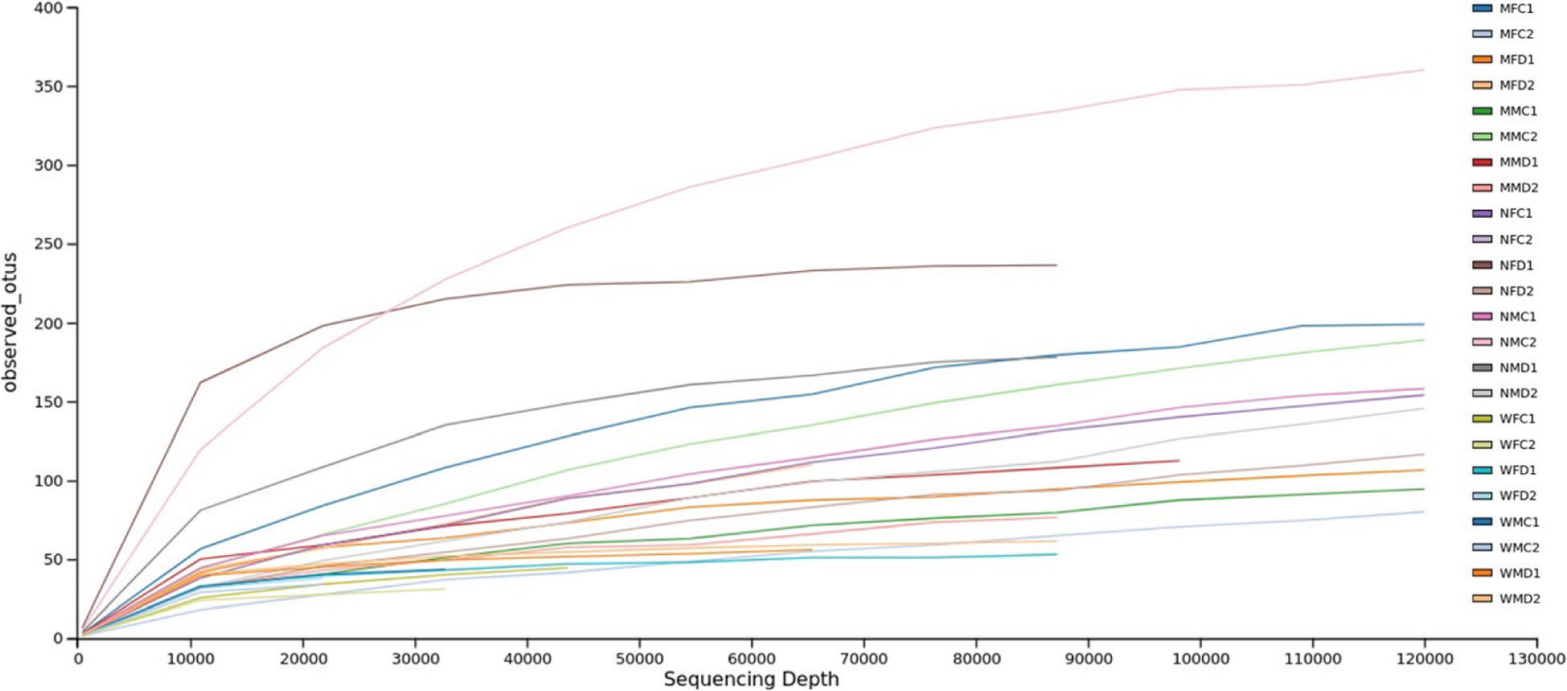
Rarefaction analysis of bacterial communities in gut of wild and mass-reared Zeugodacus cucurbitae and *Bactrocera dorsalis* adult samples. MFC1 and 2: Mature female cucurbitae; MFD1 and 2: Mature female dorsalis; MMC1 and 2: Mature male cucurbitae; MMD1 and 2: Mature male dorsalis; NFC1 and 2: Newly emerged female cucurbitae; NFD1 and 2: Newly emerged female dorsalis; NMC1 and 2: Newly emerged male cucurbitae; NMD1 and 2: Newly emerged male dorsalis; WFC1 and 2: Wild female cucurbitae; WFD 1 and 2: Wild female dorsalis; WMC1 and 2: Wild male cucurbitae; WMD1 and 2: Wild male dorsalis

We obtained an average of 122,227 high quality reads per sample in the variable regions of V3 and V4 region of *16S rRNA* gene. The total numbers of reads varied between wild male and female, also in mass-reared mature and newly emerged *Z. cucurbitae* and *B. dorsalis* adult samples. The Good’s coverage was 99.9% in all samples of both fruit fly species indicating sequencing depth (Table 1). Rarefaction analysis showed that sequences sharply increased before approaching a plateau (Fig. 1).

**Table 1:** Richness and diversity estimation of the 16S rRNA gene libraries from the metagenomic analysis of gut bacterial communities of wild and mass-reared Zeugodacus cucurbitae and *Bactrocera dorsalis* adult samples

Fruit fly samples/Diversity index	Shannon diversity	Simpson evenness	Goods coverage (%)
WFC	1.41	0.34	99.9
WMC	1.67	0.29	99.9
WFD	0.94	0.58	99.9
WMD	1.56	0.38	99.9
MFC	1.05	0.54	99.9
MMC	1.31	0.41	99.9
NFC	0.71	0.69	99.9
NMC	0.97	0.62	99.9
MFD	1.64	0.34	99.9
MMD	1.97	0.27	99.9
NFD	0.96	0.59	99.9
NMD	1.30	0.37	99.9

*Z* cucurbitae: *WFC* Wild female cucurbitae; *WMC* Wild male cucurbitae; *MFC* Mature female cucurbitae; *MMC* Mature male cucurbitae; *NFC* Newly emerged female cucurbitae; *NMC* Newly emerged male cucurbitae. *B. dorsalis*; *WFD* Wild female dorsalis; *WMD* Wild male dorsalis; *MFD* Mature female dorsalis; *MMD* Mature male dorsalis; *NFD* Newly emerged female dorsalis and *NMD* Newly emerged male dorsalis

### Bacterial diversity in gut of fruit fly species

The bacterial richness and diversity was estimated for gut of wild and mass-reared *Z. cucurbitae* and *B. dorsalis* adults. Substantial diversity was observed in both stages of wild and mass-reared mature and newly emerged *Z. cucurbitae* and *B. dorsalis* adults, although coverage estimates were very high for all samples of fruit fly (Table 1). In case of *Z. cucurbitae* adults, the Shannon and Simpson indices of bacterial diversity showed difference for wild (Shannon diversity: 1.41–1.67 Simpson evenness: 0.29–0.34), mass-reared mature (Shannon diversity: 1.05–1.31; Simpson evenness: 0.41–0.54) and newly emerged (Shannon diversity: 0.71–0.97; Simpson evenness: 0.62–0.69). Similarly, *B. dorsalis* wild (Shannon diversity: 0.94–1.56; Simpson evenness: 0.38–0.58) and mass-reared mature (Shannon diversity: 1.64–1.97; Simpson evenness: 0.27–0.34) and newly emerged (Shannon diversity: 0.96–1.30; Simpson evenness: 0.37–0.59) adults were also showed variation in bacterial diversity (Table 1).

### Relative abundance of bacterial taxa in the gut of fruit fly species

**Fig. 2:**
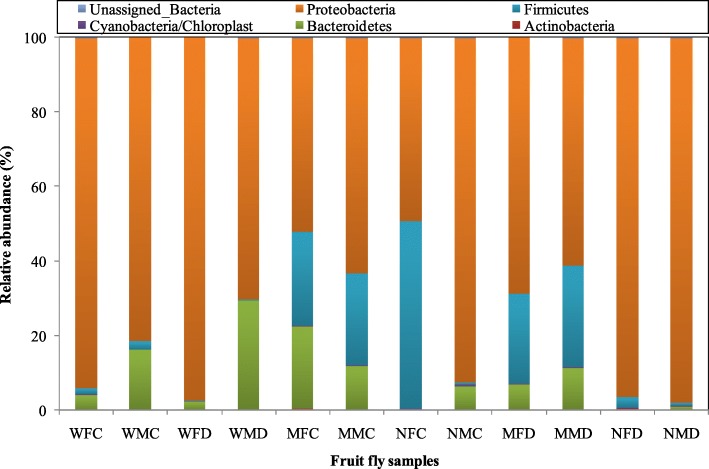
Relative abundance (%) of bacterial phyla obtained from the gut of wild and mass-reared mature and newly emerged Zeugodacus cucurbitae and *Bactrocera dorsalis* adult samples. Z. cucurbitae: WFC: Wild female cucurbitae; WMC: Wild male cucurbitae; MFC: Mature female cucurbitae; MMC: Mature male cucurbitae; NFC: Newly emerged female cucurbitae; NMC: Newly emerged male cucurbitae. B. dorsalis: WFD: Wild female dorsalis; WMD: Wild male dorsalis; MFD: Mature female dorsalis; MMD: Mature male dorsalis; NFD: Newly emerged female dorsalis and NMD: Newly emerged male dorsalis

The relative abundance of bacterial phyla significantly varied between wild and mass-reared fruit fly species (*p < 0.05*) and age (*p < 0.05)* of adult samples. Five phyla Actinobacteria, Bacteroidetes, Cyanobacteria/Chloroplast, Firmicutes, and Proteobacteria were detected and there is significant difference (*p* < 0.05) in the samples of wild and mass-reared *Z. cucurbitae* and *B. dorsalis* adults (Fig. 2). Phylum Cyanobacteria/Chloroplast was present with very low relative abundance in the wild as well as in mass-reared fruit fly species. The Phylum Proteobacteria was more abundant in wild *Z. cucurbitae* (~ 87.72%) than wild *B. dorsalis* (~ 83.87%). Similarly, this phylum was also dominant in mass-reared *Z. cucurbitae* (64.15%) and *B. dorsalis* (~ 80.96%) (Fig. 2). Among them, newly emerged fruit fly adults (83.85%) contributed Proteobacteria which shows significantly higher prevalence than to mature fruit fly adults (61.25%) (Fig. 2). The higher relative abundance of another phylum Firmicutes was observed in mass reared as compared to wild fruit fly adults. The bacterial communities belonging to Phylum Bacteroidetes was also observed in wild adults of both species in the range of 2.46–29.45%. While range of relative abundance of this phylum was more in mature adults (7.08–22.08%) than newly emerged (0.14–6.41%) of *Z. cucurbitae* and *B. dorsalis* adults. Some reads were also recovered as unassigned bacteria from both the species samples (Fig. 2). Among fruit fly adults, sex of the insect did not have an effect on bacterial phyla composition (*p > 0.05*). The relative abundance and distribution of the bacteria from wild and mass-reared mature and newly emerged fruit fly species at class and order level are shown in Additional file 1: Figure S1 A & B, respectively.

**Fig. 3:**
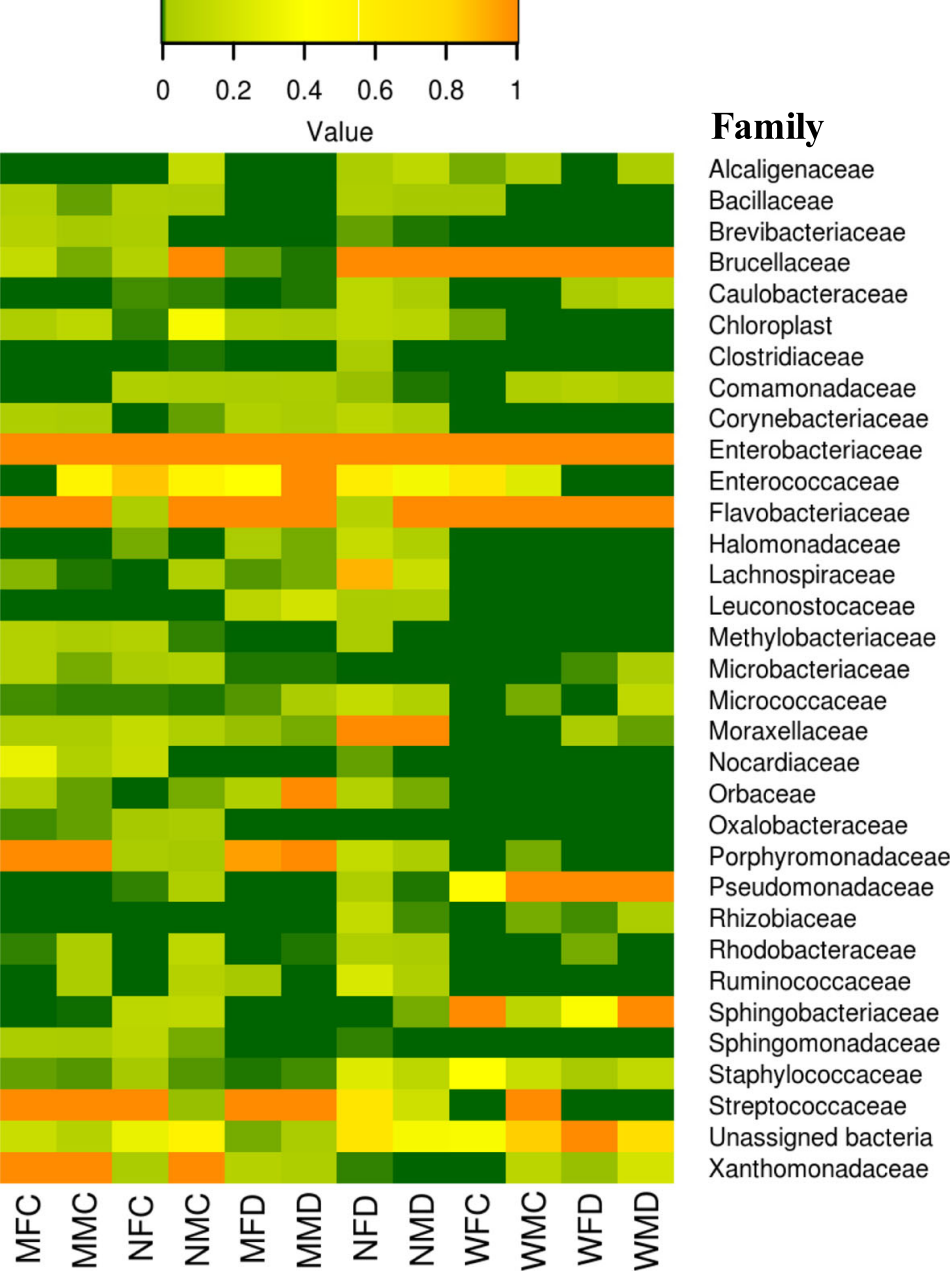
Heat maps showing relative abundance of dominant bacterial families identified from gut of wild and mass-reared mature and newly emerged *Zeugodacus cucurbitae* and *Bactrocera dorsalis* adult samples. The color code indicates relative abundance, ranging from green (low abundance) to yellow to orange (high abundance). *Z. cucurbitae*: WFC: Wild female cucurbitae; WMC: Wild male cucurbitae; MFC: Mature female cucurbitae; MMC: Mature male cucurbitae; NFC: Newly emerged female cucurbitae; NMC: Newly emerged male cucurbitae. *B. dorsalis*: WFD: Wild female dorsalis; WMD: Wild male dorsalis; MFD: Mature female dorsalis; MMD: Mature male dorsalis; NFD: Newly emerged female dorsalis and NMD: Newly emerged male dorsalis

The bacterial families present in reads significantly varied with species (*p < 0.05*) and age of the of fruit fly adults (*p < 0.05*). Among the families, Enterobacteriaceae was detected in all samples of fruit fly species. This family was more predominant in wild *Z. cucurbitae* adults (68.77%) as compared to mature (54.23%) and newly emerged (46.22%), whereas, Enterobacteriaceae was less abundant in wild *B. dorsalis* (53.22%) than mature (63.60%) and newly emerged (81.12%) mass-reared adults (Fig. 3). Streptococcaceae and Flavobacteriaceae were other predominant families in wild as well as mass-reared *Z. cucurbitae* and *B. dorsalis* samples (Fig. 3). Pseudomonadaceae and Sphingobacteriaceae families were only detected in wild fruit fly adults of both species. Family Brucellaceae showed higher relative abundance in mass-reared newly emerged *Z. cucurbitae* as compared to wild and mass-reared matured adults. Moraxellaceae represented considerable level of relative abundance in newly emerged *B. dorsalis* than *Z. cucurbitae*. The relative abundance of Porphyromonadaceae family was more in mass-reared matured adults as compared to newly emerged adults.

**Fig. 4:**
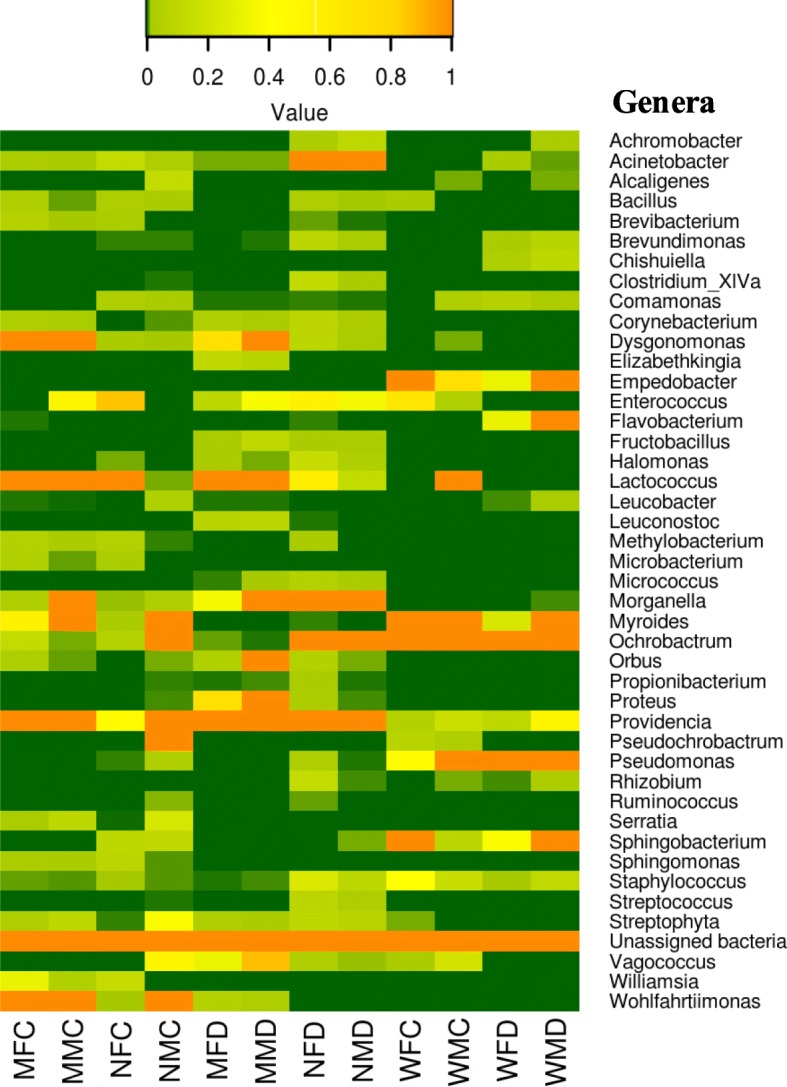
Heat maps showing relative abundance of dominant bacterial genera identified from gut of wild and mass-reared mature and newly emerged *Zeugodacus cucurbitae* and *Bactrocera dorsalis* adult samples. The color code indicates relative abundance, ranging from green (low abundance) to yellow to orange (high abundance). *Z. cucurbitae*: WFC: Wild female cucurbitae; WMC: Wild male cucurbitae; MFC: Mature female cucurbitae; MMC: Mature male cucurbitae; NFC: Newly emerged female cucurbitae; NMC: Newly emerged male cucurbitae. *B. dorsalis*: WFD: Wild female dorsalis; WMD: Wild male dorsalis; MFD: Mature female dorsalis; MMD: Mature male dorsalis; NFD: Newly emerged female dorsalis and NMD: Newly emerged male dorsalis

Genus *Providencia* (Enterobacteriaceae) was present in wild as well as mass-reared fruit fly species adults. This genus was dominant in mass-reared newly emerged adults (ZC:~ 15.41%; BD: ~ 33.68%) than mass-reared matured (ZC: ~ 4.73%; BD: ~ 2.70%) fruit fly samples (Fig. 4). *Lactococcus* is another genus frequently occurred in mass-reared fruit fly adults except newly emerged males of *Z. cucurbitae* samples (24.60%). While, genus *Acinetobacter* was represented strongly in newly emerged male and females of both the species but occurred in low relative abundance in mature adults (Fig. 4). Other genera like *Ochrobactrum*, *Myroides*, *Vagococcus*, *Corynebacterium, Staphylococcus* and *Enterococcus* were also detected with lower relative abundance in both wild as well as in mass-reared fruit fly adults. While, *Dysgonomonas* and *Wohlfahrtiimonas* were only present in mass-reared fruit fly species adults (Fig. 4). *Morganella* and *Serratia* genera were also detected more in mass-reared mature and newly emerged *B. dorsalis* adults (Fig. 4). Overall, the bacterial genera showed a significant variation in relative abundance (*p* < 0.05) in wild and mass-reared fruit fly adults. The number of bacterial species and their relative abundance showed significant differences (*p* < 0.05) while no significant difference between fruit fly species (*p* > 0.05) and age of the insects (*p* > 0.05) was observed (Additional file 2: Table S1). The relative abundance of *Empedobacter brevis*, *Myroides odoratus* and *Sphingobacterium yanglingense* bacterial species were more prevalent in wild *Z. cucurbitae* and *B. dorsalis* adults (Additional file 2: Table S1). *Myroides marinus*, *Dysgonomonas capnocytophagoides* and *Wohlfahrtiimonas larvae* species were identified in mass-reared mature adults of both the species. While, *Acinetobacter baumannii* (8.9–15.7%) occurred in mass-reared newly emerged *B. dorsalis* adults.

**Fig. 5:**
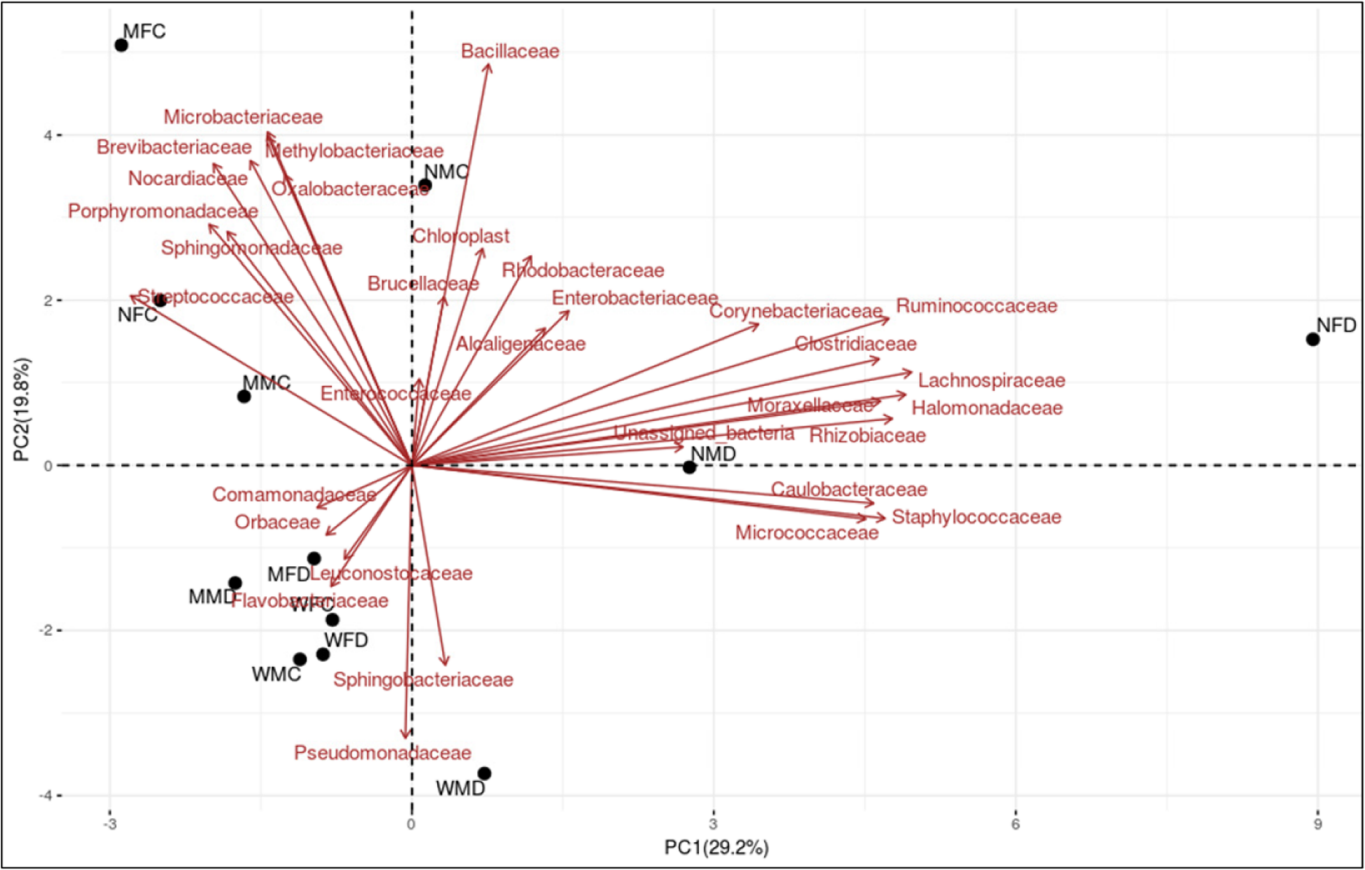
Comparison of bacterial communities in wild and mass-reared *Zeugodacus cucurbitae* and *Bactrocera dorsalis* adult samples. Principal Coordinate Analysis (PCoA) was generated with bacterial composition of the families among wild and mass-reared fruit fly species. *Z. cucurbitae*: WFC: Wild female cucurbitae; WMC: Wild male cucurbitae; MFC: Mature female cucurbitae; MMC: Mature male cucurbitae; NFC: Newly emerged female cucurbitae; NMC: Newly emerged male cucurbitae. *B. dorsalis*: WFD: Wild female dorsalis; WMD: Wild male dorsalis; MFD: Mature female dorsalis; MMD: Mature male dorsalis; NFD: Newly emerged female dorsalis and NMD: Newly emerged male dorsalis

Overall relative abundance of bacterial communities present in the gut of wild as well as in mass-reared fruit fly species are significantly different. This indicates that the diet used during mass-rearing of insects significantly influences the bacterial taxa composition (*p* < 0.05). Sex of the insect did not have an effect on bacterial taxa (*p > 0.05*). Moreover, PCoA also confirmed that fruit fly adult samples were grouped based on fruit fly species and age of the adult i.e. wild and mass-reared (Fig. 5). However, no such grouping was formed on the basis of sex of the adult.

## Discussion

The success of sterile insect technique (SIT) under field condition is greatly influenced by the fitness of sterile insects. The insect gut bacteria are known to diversified due to domestication, diet, colony management and irradiation [8, 20]. Probiotic application of insect gut bacteria could enhance the fitness of sterile insects [7–11]. We used next generation sequencing technique to illustrate the gut bacterial composition of wild and mass-reared melon fly and Oriental fruit fly adults. The results indicate that significant difference was observed in relative abundance of bacterial communities in adults of *Z. cucurbitae* and *B. dorsalis* obtained from wild and mass-rearing facility*.* Four phyla Actinobacteria, Bacteroidetes, Firmicutes and Proteobacteria were occurred in all the samples of *Z. cucurbitae* and *B. dorsalis* adults. Phyla Actinobacteria, Bacteroidetes, Firmicutes and Proteobacteria are frequently recovered from different development stages of *Bactrocera* sp. [32, 34, 35]. Our results showed that Proteobacteria and Firmicutes are major phyla in wild as well as in mass-reared *Z. cucurbitae* and *B. dorsalis* adult samples. Proteobacteria was more represented in gut of wild as compared to mass-reared *Z. cucurbitae* and *B. dorsalis* adults. While, the relative abundance of Phylum Firmicutes was higher in mass reared insects as compared to wild fruit fly adults. It has been reported that these phyla are dominant in the gut of *Bactrocera* species adults like Chinese citrus fly (*Bactrocera minax* (Enderlein)), Carambola fruit fly (*B. carambolae* Drew & Hancock) and *B. dorsalis* [32–34]. Actinobacteria and Bacteroidetes are also present in both *Zeugodacus* and *Bactrocera* sp. with varied relative abundance. Low level abundance of Bacteroidetes occurred in wild and mass reared mature *Z. cucurbitae* and *B. dorsalis* adults, while, Actinobacteria was observed only in newly emerged mass-reared adults. The occurrence of these phyla has been documented in *B. dorsalis* and *B. carambolae* [32–34]. It has been observed that Actinobacteria influences various metabolic and physiological activities including extracellular enzyme production, antimicrobial activity and other secondary metabolites formation [36, 37]. Thus, this phylum may be essential for contributing in growth and development of *Z. cucurbitae* and *B. dorsalis*.

In the present study, Enterobacteriaceae (Proteobacteria-Gammaproteobacteria), Streptococcaceae (Firmicutes-Bacilli) and Flavobacteriaceae (Bacteroidetes-Flavobacteriia) were dominant families in both fruit fly species and the abundance varied with origin and age of the adults. Members of these families are known to be dominant and frequently identified from the gut of *Bactrocera* sp. [15–17, 19, 32–35]. The presence of Enterobacteriaceae in wild and mass-reared insects suggests important role of this family in insect growth and development [8].

*Providencia* (Enterobacteriaceae) occurred in wild as well as mass-reared adults followed by *Lactococcus* (*Streptococcaceae*) which is merely prevalent in wild fruit fly adults. This genus has been isolated from several fruit flies species including *Anastrepha ludens* (Loew), *B. dorsalis*, *B. cucurbitae*, *B. minax* and *Bactrocera tau* (Walker) [15–17, 23, 32, 33] and known to regulate the growth of other bacteria in the gut [38]. Other genera like *Ochrobactrum*, *Myroides*, *Vagococcu*s, *Corynebacterium, Staphylococcus* and *Enterococcus* were observed in both wild as well as mass-reared fruit fly adults. This suggests the gut bacterial taxa significantly differ with fruit fly species, origin and age of the adults. Similarly, it has been also evident that different bacterial composition was observed among conspecific and interspecific *B. carambolae* and *B. dorsalis* samples through metagenomic analysis [34]. The deleterious microorganisms like *Morganella* and *Serratia* were detected only in mass-reared *Z. cucurbitae* and *B. dorsalis* adults. These genera are known to colonise in different mass-reared insects including fruit fly colonies [25, 32], this may be due to lower occurrence of certain members of Enterobacteriaceae which are known to prevent proliferation of pathogenic bacteria and also indirectly contributing in to host fitness [8, 11, 12]. Further, it has been observed that changes in the relative proportion of the different bacterial communities in the guts of mass reared medflies of the Vienna-8 strain [8].

The gut bacterial taxa significantly differed with the diet of fruit fly species. The specific natural diets used in the present study might have influenced the bacterial composition in wild and mass-reared fruit fly species and may play an important role in growth and development of insects. Some bacterial communities are more abundant in newly emerged adults as compared to wild and mass-reared mature adults. It has been observed that diet of insects and surroundings during mass rearing significantly influences the composition of gut microbiota [8, 11, 20, 39].

The influence of sex of the insects on the bacterial composition is minimal in both the fruit fly species in the present study. PCoA analysis also revealed that no groups were formed based on sexes of the species. Similarly, minimal variation in bacterial communities was observed in *B. minax* sexes, while significantly different bacterial communities were identified between the gut and the reproductive organs [32]. It has also been documented in medflies of the Vienna-8 strain that gut bacterial diversity varies with life stage and the sex of the insect, however, overall bacterial composition structure was not affected [11]. The present study showed the composition of bacterial communities in the gut of wild and mass-reared melon fly and Oriental fruit fly adults from India. The bacterial taxa were influenced by diet, varied with species and age but not with sex of the insects.

## Conclusions

Gut bacterial diversity of wild and mass-reared *Z. cucurbitae* and *B. dorsalis* was analyzed using metagenomic sequencing of *16S rRNA* gene. Diversity of the inhabited bacterial communities from wild and mass-reared fruit fly adults was compared. Significant difference was observed in relative abundance of bacterial communities in the adults of *Z. cucurbitae* and *B. dorsalis* obtained from wild and mass-rearing facility*.* The most representative phyla were Actinobacteria, Bacteroidetes, Firmicutes and Proteobacteria in all samples of *Z. cucurbitae* and *B. dorsalis* adults. Among bacterial families, Enterobacteriaceae was predominant in the gut of wild and mass-reared adults of fruit fly. Genera *Providencia* and *Lactococcus* were dominant and present in all samples of the fruit fly with varied level of relative abundance. The PCoA analysis showed a distinct grouping of fruit fly species and age of the adults (wild, mature or newly emerged) while no grouping was formed based on the sex of the adults. Further studies will be focussed on identifying function of representative species for either pathogenic or probiotic activity of gut bacterial species. This will help in designing efficient mass rearing protocols for successful implementation of SIT in the field.

## Methods

### Fruit fly sample collection

The melon fly (*Z. cucurbitae*) infested pumpkin (*Cucurbita* sp.) were collected from cucurbits farm from Gouribidanur region of Karnataka state in India (13° 62′ N and 77°51′ E) while Oriental fruit fly (*B. dorsalis*) infested mango (*Mangifera indica* L.) were collected from Dahanu region of Maharashtra state in India (19^o^ 97′ N and 72^o^ 73′ E). The infested fruits were brought to the laboratory and placed in sterile sand until pupation. Upon pupation, pupae were placed individually in sterile vials (5 mL capacity) for adult emergence. These newly emerged adults of both the species are mentioned as ‘wild’ throughout the text. The wild adults emerged from pupae of both the species were used for gut extraction and further designated as follows: wild female *Z.* *cucurbitae* (WFC), wild male *Z.* *cucurbitae* (WMC) and wild female *B.* *dorsalis* (WFD) and wild male *B.* *dorsalis* (WMD) (Table 2). In case of laboratory reared (mass-reared), the melon fly (> 36 generations) and Oriental fruit fly (> 30 generations) cultures were maintained on bottle gourd (*Lagenaria siceraria* (Molina) Standley) and sweet banana (*Musa* sp.) at 28 ± 2 °C and 75% RH, respectively at insect rearing facility, Bhabha Atomic Research Centre (BARC), Mumbai, India. Adult flies were reared in two-side fine mesh cages (45 × 45 × 45 cm) and provided with ProtineX (Nutricia International Private Ltd., Mumbai, India) and water. Upon pupation, pupae were placed individually in sterile vials (5 mL capacity) for adult emergence. The mature (~ 12 days old) (M) and newly emerged (N) (< 1 h after emergence) male and female adults of *Z. cucurbitae* and *B. dorsalis* were used for gut extraction and further designated as follows: mature female of *Z.* *cucurbitae* (MFC), mature male of *Z.* *cucurbitae* (MMC), newly emerged female of *Z.* *cucurbitae* (NFC); newly emerged male of *Z.* *cucurbitae* (NMC); mature female of *B.* *dorsalis* (MFD); mature male of *B.* *dorsalis* (MMD); newly emerged female of *B.* *dorsalis* (NFD) and newly emerged male of *B.* *dorsalis* (NMD) (Table 2).

**Table 2:** The melon fly (Zeugodacus cucurbitae) and Oriental fruit fly (*Bactrocera dorsalis*) adult populations used in this study

Wild or mass-reared adults	Accession code	Host	Location	Number of samples^a^
*Z. cucurbitae*	WFC	*Cucurbita* sp.	Gouribidanur, Karnataka, India	30
	WMC	*Cucurbita* sp.	Gouribidanur, Karnataka, India	30
*B. dorsalis*	WFD	*Mangifera indica*	Dahanu, Maharashtra, India	30
	WMD	*Mangifera indica*	Dahanu, Maharashtra, India	30
*Z. cucurbitae*	MFC	*Lagenaria siceraria*	Insect rearing facility, BARC, Mumbai, India	30
	MMC	*Lagenaria siceraria*	Insect rearing facility, BARC, Mumbai, India	30
	NFC	*Lagenaria siceraria*	Insect rearing facility, BARC, Mumbai, India	30
	NMC	*Lagenaria siceraria*	Insect rearing facility, BARC, Mumbai, India	30
*B. dorsalis*	MFD	*Musa* sp.	Insect rearing facility, BARC, Mumbai, India	30
	MMD	*Musa* sp.	Insect rearing facility, BARC, Mumbai, India	30
	NFD	*Musa* sp.	Insect rearing facility, BARC, Mumbai, India	30
	NMD	*Musa* sp.	Insect rearing facility, BARC, Mumbai, India	30

*Z* cucurbitae: *WFC* Wild female cucurbitae; *WMC* Wild male cucurbitae; *MFC* Mature female cucurbitae; *MMC* Mature male cucurbitae; *NFC* Newly emerged female cucurbitae; *NMC* Newly emerged male cucurbitae. *B. dorsalis*; *WFD* Wild female dorsalis; *WMD* Wild male dorsalis; *MFD* Mature female dorsalis; *MMD* Mature male dorsalis; *NFD* Newly emerged female dorsalis and *NMD*: Newly emerged male dorsalis.

^a^Pooled DNA of fifteen adult samples of male/female as one replicate and two replicates were used for both species in this study

### Fruit fly dissection and DNA extraction

The wild and mass-reared male and female adults of melon fly and Oriental fruit fly were surface sterilized by submerging them sequentially in 70% ethanol for 1 min, 0.5% sodium hypochlorite for 1 min and washed twice (1 min each) in sterile distilled water [16, 19]. Total thirty male and female of wild and mass-reared adults of both species were used in this study. The surface sterilized flies were individually dissected (*n* = 15 of each adult species/replicate; two replicates) aseptically under a clean air workstation. The insect midgut portion extending from the crop to the joints of malpighian tubules were separated and transferred to a sterile 1.5 mL microcentrifuge tube. Total genomic DNA was extracted from the pooled gut content obtained from *Z. cucurbitae* and *B. dorsalis* samples by using PureLink Genomic DNA kit (Invitrogen, Germany) according to manufacturer’s instructions. The quantity and quality of DNA was determined by NanoDrop 2000 (Thermo Scientific, USA) spectrophotometric analysis at 260 nm and agarose gel (0.8%) electrophoresis.

### PCR, Illumina sequencing and data processing

Bacterial communities present in the gut of wild and mass-reared adults of *Z. cucurbitae* and *B. dorsalis* were studied using Illumina HiSeq next-generation sequencing (NGS) of *16S rRNA* gene. Polymerase chain reaction (PCR) was performed as described with PCR Protocol for Phusion® High-Fidelity DNA Polymerase (New England Biolabs, UK). We used Pro341F (5′- CCTACGGGNBGCASCAG-3′) and Pro805R (5′-GACTACNVGGGTATCTAATCC-3′) primers to amplify the V3-V4 regions of the *16S rRNA* gene [40]. PCR products were further purified using PureLink® PCR Purification Kit (Invitrogen, Germany). Sequencing was conducted using a paired-end, 2 × 250-bp cycle run on an Illumina HiSeq Rapid V2 kit (AgriGenome Labs Pvt. Ltd., Kochi, India).

Demultiplexed raw sequences were extracted from the Illumina HiSeq system in FASTQ format and the quality of sequences was evaluated using the bcl2fastQ software (Illumina Inc). The reads were pre-processed in UPARSE implemented in USEARCH 10.0.240 [41]. We have used the maximum overlap 150 bp and minimum overlap of 30 bp to get all possible consensuses. While making consensus V3-V4 sequences, the passed reads were further aligned to each other with a contig length of 400–480 bp. The first step was merging paired reads of all samples into a single FASTQ file. The primers were stripped and the filtered sequences were obtained. The reads with >Q30 were included in the analysis. Further, singletons were discarded and unique and abundant sequences identified using USEARCH [42]. Chimeras were removed using cluster_otus command implemented in the tool USEARCH [37]. The sequences were subsequently clustered into operational taxonomic units (OTUs) at a 97% similarity level. Representative sequence and taxonomy classification was identified for each OTU and aligned against the Ribosomal Database Project (RDP) training set of sequences (http://www.drive5.com/sintax/rdp_16s_v16_sp.fa.gz). QIIME program (QIIME 2–2018.8) was employed for the further downstream analysis by using OTU table after converting to the Biological Observation Matrix (BIOM) format [43]. The alpha diversity indices and rarefaction analysis were obtained using QIIME (2–2018.8). Further, heat maps [44] were generated from relative abundance of family and genera while Principal Coordinate Analysis (PCoA) plot was obtained from bacterial composition of families implemented in R program ver. 3.5. (https://www.R-project.org/). We used nonparametric Kruskal-Wallis tests in R v. 3.4.1 to determine whether there were significant differences in the relative abundances of bacterial taxa (phylum to species) in fruit fly adult samples. We selected all the bacterial taxa which were contributing at least > 0.01% of relative abundance in any one of study groups.

## Supplementary information

**Additional file 1: Figure S1.** Relative abundance (%) of major bacterial classes (A) and orders (B) identified in the gut of wild and mass-reared *Zeugodacus cucurbitae* and *Bactrocera dorsalis* adult samples revealed by metagenomic analysis. *Z. cucurbitae*: WFC: Wild female cucurbitae; WMC: Wild male cucurbitae; MFC: Mature female cucurbitae; MMC: Mature male cucurbitae; NFC: Newly emerged female cucurbitae; NMC: Newly emerged male cucurbitae. *B. dorsalis*: WFD: Wild female dorsalis; WMD: Wild male dorsalis; MFD: Mature female dorsalis; MMD: Mature male dorsalis; NFD: Newly emerged female dorsalis and NMD: Newly emerged male dorsalis.

**Additional file 2: Table S1.** Relative abundance (%) of major bacterial species (with > 1% in at least one adult sample) in gut of wild and mass-reared *Zeugodacus cucurbitae* and *Bactrocera dorsalis* adult samples.

## Data Availability

The data that support the findings of this study are available from the corresponding author upon reasonable request.

## Abbreviations

BD: *Bactrocera dorsalis*
BIOM: Biological Observation Matrix
DGGE: Denaturing gradient gel electrophoresis
NGS: Next-generation sequencing
OTU: Operational taxonomic units
PCoA: Principal Coordinate Analysis
SIT: Sterile insect technique
ZC: Zeugodacus cucurbitae
