# Supplementary material for: Bacterial communities in the gut of wild and mass-reared Zeugodacus cucurbitae and Bactrocera dorsalis revealed by metagenomic sequencing
Source: BMC Microbiol. 2019 Dec 24;19(Suppl 1):282. doi: 10.1186/s12866-019-1647-8 (PMC6929459; doi:10.1186/s12866-019-1647-8)
Supplement: Supplementary file 1 — Additional file 1: Figure S1. Relative abundance (%) of major bacterial classes (A) and orders (B) identified in the gut of wild and mass-reared Zeugodacus cucurbitae and Bactrocera dorsalis adult samples revealed by metagenomic analysis. Z. cucurbitae: WFC: Wild female cucurbitae; WMC: Wild male cucurbitae; MFC: Mature female cucurbitae; MMC: Mature male cucurbitae; NFC: Newly emerged female cucurbitae; NMC: Newly emerged male cucurbitae. B. dorsalis: WFD: Wild female dorsalis; WMD: Wild male dorsalis; MFD: Mature female dorsalis; MMD: Mature male dorsalis; NFD: Newly emerged female dorsalis and NMD: Newly emerged male dorsalis. [file 12866_2019_1647_MOESM1_ESM.doc]

**Additional file 1: Figure S1.** Relative abundance (%) of major bacterial classes (A) and orders (B) identified in the gut of wild and mass-reared *Zeugodacus cucurbitae* and *Bactrocera dorsalis* adult samples revealed by metagenomic analysis. *Z. cucurbitae*: WFC: Wild female cucurbitae; WMC: Wild male cucurbitae; MFC: Mature female cucurbitae; MMC: Mature male cucurbitae; NFC: Newly emerged female cucurbitae; NMC: Newly emerged male cucurbitae. *B. dorsalis*: WFD: Wild female dorsalis; WMD: Wild male dorsalis; MFD: Mature female dorsalis; MMD: Mature male dorsalis; NFD: Newly emerged female dorsalis and NMD: Newly emerged male dorsalis.

**A)**

**B)**
