# Supplementary material for: Bacterial communities in the gut of wild and mass-reared Zeugodacus cucurbitae and Bactrocera dorsalis revealed by metagenomic sequencing
Source: BMC Microbiol. 2019 Dec 24;19(Suppl 1):282. doi: 10.1186/s12866-019-1647-8 (PMC6929459; doi:10.1186/s12866-019-1647-8)
Supplement: Supplementary file 2 — Additional file 2: Table S1. Relative abundance (%) of major bacterial species (with > 1% in at least one adult sample) in gut of wild and mass-reared Zeugodacus cucurbitae and Bactrocera dorsalis adult samples. [file 12866_2019_1647_MOESM2_ESM.doc]

**Additional file 2: Table S1.** Relative abundance (%) of major bacterial species (with >1% in at least one adult sample) in gut of wild and mass-reared *Zeugodacus cucurbitae* and *Bactrocera dorsalis* adult samples.

| **Bacterial species** | **WFC**  **± SE** | **WMC**  ± **SE** | **WFD**  ± **SE** | **WMD**  ± **SE** | **MFC**  ± **SE** | **MMC**  ± **SE** | **NFC**  ± **SE** | **NMC**  ± **SE** | **MFD**  ± **SE** | **MMD**  ± **SE** | **NFD**  ± **SE** | **NMD**  ± **SE** |
| --- | --- | --- | --- | --- | --- | --- | --- | --- | --- | --- | --- | --- |
| *Acinetobacter baumannii* | 0.0±0.0 | 0.0±0.0 | 0.0±0.0 | 0.0±0.0 | 0.0±0.0 | 0.0±0.0 | 0.0±0.0 | 0.0±0.0 | 0.0±0.0 | 0.0±0.0 | 15.7±15.7 | 8.9±8.9 |
| *Dysgonomonas*  *capnocytophagoides* | 0.0±0.0 | 0.0±0.0 | 0.0±0.0 | 0.0±0.0 | 1.6±0.1 | 1.2±1.0 | 0.0±0.0 | 0.0±0.0 | 0.0±0.0 | 1.7±1.5 | 0.0±0.0 | 0.0±0.0 |
| *Empedobacter brevis* | 1.1±0.8 | 0.7±0.7 | 0.3±0.0 | 7.4±6.3 | 0.0±0.0 | 0.0±0.0 | 0.0±0.0 | 0.0±0.0 | 0.0±0.0 | 0.0±0.0 | 0.0±0.0 | 0.0±0.0 |
| *Flavobacterium ceti* | 0.0±0.0 | 0.0±0.0 | 0.3±0.2 | 1.4±0.4 | 0.0±0.0 | 0.0±0.0 | 0.0±0.0 | 0.0±0.0 | 0.0±0.0 | 0.0±0.0 | 0.0±0.0 | 0.0±0.0 |
| *Myroides marinus* | 0.0±0.0 | 0.2±0.2 | 0.0±0.0 | 0.0±0.0 | 0.6±0.6 | 5.7±5.7 | 0.0±0.0 | 0.2±0.2 | 0.0±0.0 | 0.0±0.0 | 0.0±0.0 | 0.0±0.0 |
| *Myroides odoratus* | 1.0±1.0 | 15.2±15.2 | 0.2±0.2 | 2.9±2.8 | 0.0±0.0 | 0.0±0.0 | 0.0±0.0 | 5.7±5.7 | 0.0±0.0 | 0.0±0.0 | 0.0±0.0 | 0.0±0.0 |
| *Proteus hauseri* | 0.0±0.0 | 0.0±0.0 | 0.0±0.0 | 0.0±0.0 | 0.0±0.0 | 0.0±0.0 | 0.0±0.0 | 0.0±0.0 | 0.7±0.3 | 1.5±0.8 | 0.0±0.0 | 0.0±0.0 |
| *Sphingobacterium*  *yanglingense* | 2.3±2.3 | 0.1±0.1 | 0.0±0.0 | 0.1±0.0 | 0.0±0.0 | 0.0±0.0 | 0.1±0.1 | 0.1±0.1 | 0.0±0.0 | 0.0±0.0 | 0.0±0.0 | 0.0±0.0 |
| *Wohlfahrtiimonas larvae* | 0.0±0.0 | 0.0±0.0 | 0.0±0.0 | 0.0±0.0 | 2.6±2.6 | 3.7±3.7 | 0.0±0.0 | 1.5±1.5 | 0.1±0.0 | 0.0±0.0 | 0.0±0.0 | 0.0±0.0 |

SE: Standard error of two replicates: *Z. cucurbitae*: WFC: Wild female cucurbitae; WMC: Wild male cucurbitae; MFC: Mature female cucurbitae; MMC: Mature male cucurbitae; NFC: Newly emerged female cucurbitae; NMC: Newly emerged male cucurbitae. *B. dorsalis*: WFD: Wild female dorsalis; WMD: Wild male dorsalis; MFD: Mature female dorsalis; MMD: Mature male dorsalis; NFD: Newly emerged female dorsalis and NMD: Newly emerged male dorsalis.
